# The impact of breaking up prolonged sitting on glucose metabolism and cognitive function when sleep is restricted

**DOI:** 10.1016/j.nbscr.2017.09.001

**Published:** 2017-09-14

**Authors:** Grace E. Vincent, Sarah M. Jay, Charli Sargent, Katya Kovac, Corneel Vandelanotte, Nicola D. Ridgers, Sally A. Ferguson

**Affiliations:** a Central Queensland University, Health, Medical and Applied Sciences, Wayville 5034, Australia; b Deakin University, Institute of Physical Activity and Nutrition (IPAN), School of Exercise and Nutrition Sciences, Geelong, Australia

**Keywords:** Physical activity, Exercise, Sitting breaks, Sedentary behaviour, Fatigue, Sleepiness

## Abstract

**Objectives:**

To investigate the acute benefits of breaking up prolonged sitting with light-intensity physical activity on (i) glucose metabolism under conditions of sleep restriction, and (ii) cognitive deficits associated with sleep restriction.

**Methods:**

This counterbalanced, crossover trial consisted of two five-day (5 night) experimental conditions separated by a two-week washout period. On the first night, participants were given a 9-h sleep opportunity to allow the collection of steady-state baseline measures the following day. This was followed by three consecutive nights of sleep restriction (5-h sleep opportunity). In the sitting condition (SIT), participants remained seated between 1000 and 1800 h. In the physical activity condition (ACT), participants completed 3-min bouts of light-intensity walking every 30 min on a motorised treadmill between 1000 and 1800 h. At all other times, in both conditions, participants remained seated, except when walking to the dining room or to use the bathroom (max distance = 32 m). Six physically inactive, healthy males were randomised to one of two trial orders, 1) SIT then ACT, or 2) ACT then SIT. Continuous measures of interstitial glucose were measured at 5-min intervals. A cognitive and subjective test battery was administered every two hours during wake periods. Analyses were conducted using a series of linear mixed-effect ANOVAs.

**Results:**

No differences in interstitial glucose concentration or cognitive performance were observed between the SIT condition and the ACT condition. Participants reported higher levels of sleepiness, and felt less alert in the SIT condition compared with the ACT condition.

**Conclusions:**

There were no observable benefits of breaking up prolonged sitting on glucose metabolism under conditions of sleep restriction. These findings have implications for behaviour change interventions. Future studies will need to include larger, less homogenous study populations and appropriate control conditions (i.e., 8–9 h sleep opportunities).

## 1 Introduction

To reduce the risk factors associated with cardiometabolic disease, research and public health interventions have traditionally centred upon improving physical activity and diet, and reducing tobacco use and alcohol intake (Cannon, 2007). However, there are other important modifiable risk factors for cardiometabolic disease that have received comparatively little attention, such as prolonged sitting (Bauman et al., 2013) and inadequate sleep (Schmid et al., 2015). Determining the optimal composition of a 24-h period to promote health and prevent chronic disease represents a new direction of behaviour change intervention research (Chastin et al., 2015, Tremblay et al., 2016), beyond conventional approaches that advocate improving any single behaviour in isolation (e.g., increasing amount of moderate-vigorous physical activity). Thus, investigating how cardiometabolic disease risk factors interact with each other when they are combined is critical (e.g., prolonged sitting when sleep restricted).

Cross-sectional and prospective observational studies have indicated that prolonged sitting is a risk factor for diabetes, cardiovascular disease (CVD), and increased all-cause mortality (Wilmot et al., 2012), independent of physical activity levels (e.g., moderate-vigorous intensity physical activity such as brisk walking/jogging/sports) (Bankoski et al., 2011, Koster et al., 2012, Thorp et al., 2011, Wijndaele et al., 2014). However, a number of studies have demonstrated that breaking up prolonged sitting every 30 min, with 2–3 min of standing or short bouts of light-intensity physical activity, is associated with an improved metabolic profile (Chastin et al., 2015), reduced self-reported fatigue (Wennberg et al., 2016), and reduced all-cause mortality risk (Katzmarzyk, 2014). In addition, regularly breaking up prolonged sitting with short (1 min 40 s) bouts of light-intensity physical activity (Peddie et al., 2013) or standing (Benatti et al., 2017) is more effective than a single, continuous 30-min bout of moderate-vigorous physical activity in lowering postprandial glucose and insulin concentrations in healthy, normal weight adults. While there is good evidence that breaking up prolonged sitting is beneficial for cardiometabolic health, studies have not controlled for prior sleep duration, which is also a cardiometabolic disease risk factor.

To maintain optimal health and functioning, a typical adult should obtain at least 7 h of sleep per night (Watson et al., 2015). However, as many as 45% of adults do not meet this sleep duration recommendation (Adams et al., 2016, Centers for Disease Control Prevention, 2011). Inadequate sleep is associated with CVD, weight gain, obesity, inflammation, diabetes, and mortality (Knutson, 2010). A prospective study of healthy adults found that those who slept ≤ 6 h per night had a 15% higher risk of CVD compared with those who slept 7–8 h per night (Hoevenaar-Blom et al., 2011). Numerous well-controlled laboratory studies have observed impaired glucose metabolism with varying degrees of sleep loss (Broussard et al., 2012, Buxton et al., 2010, Nedeltcheva et al., 2009). For example, insulin sensitivity and disposition index (a marker of diabetes risk) were significantly impaired in individuals that were chronically sleep restricted (5 nights of 4 h time in bed per night) compared to those in the rested condition (5 nights of 12 h time in bed per night) (Spiegel et al., 1999). While there has been a recent move towards interventions that target breaking up prolonged sitting (e.g., implementing standing desks), any cardiometabolic benefit may be offset by sleep restriction. In essence, sleep restricted individuals may not benefit from breaking up prolonged sitting throughout the day. As a first step in exploring this hypothesis, the primary aim of this study was to determine the effects of breaking up prolonged sitting (with light-intensity physical activity) on acute cardiometabolic health outcomes under conditions of sleep restriction.

In addition to the effects on cardiometabolic health, breaking up prolonged sitting may also benefit other aspects of waking function, such as cognitive performance. Restricted sleep can result in multiple neuro-behavioural deficits, including lapses in attention, slow working memory, reduced cognitive throughput, and depressed mood (Belenky et al., 2003, Van Dongen et al., 2003). Beneficial effects of exercise on cognitive performance have been observed following a single bout of exercise (Chang et al., 2015), suggesting that at least light-intensity physical activities may mediate pathways involved in mental fatigue and cognition. Indeed, a recent pilot study found that breaking up prolonged sitting with light-intensity walking breaks may be an effective acute fatigue countermeasure, though sleep duration prior to experimentation was not reported (Wennberg et al., 2016). As such, it is unknown whether physical activity can counteract the adverse impact of sleep restriction on cognitive function by acting as a fatigue countermeasure. Therefore, a secondary aim of this pilot study is to determine whether breaking up prolonged sitting with light-intensity walking breaks can counteract the acute cognitive deficits associated with sleep restriction.

## 2 Methods

### 2.1 Study design

**Fig. 1:**
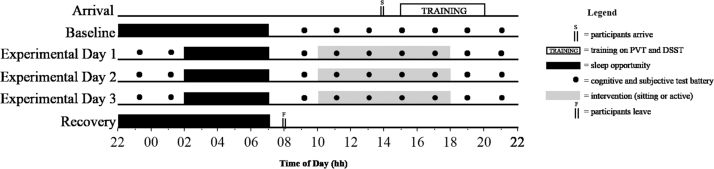
Experimental protocol.

The study was a laboratory-based, randomised, counter-balanced, crossover trial with two experimental conditions – a sitting condition (SIT) and an active condition (ACT). Participants were required to attend the laboratory on two occasions separated by a 2-week washout period. On each occasion, participants lived in the laboratory for five consecutive days and nights. An overview of the experimental protocol is shown in Fig. 1.

### 2.2 Participants

Healthy adult males (n = 6) were recruited from the Adelaide (Australia) region. Participant characteristics are reported in Table 1. Participation was voluntary and ethical approval was obtained from the Human Research Ethics Committee of Central Queensland University (H16/11-298). Participants provided written consent and were compensated financially for their time at the conclusion of the study (AU$1200).

**Table 1:** Participant demographics.

Age (y)	27.0 ± 3.7
Body mass (kg)	80.5 ± 8.7
Height (m)	1.8 ± 0.1
Waist Circumference (cm)	84.3 ± 6.7
Pittsburgh Sleep Quality Index	3.2 ± 1.9
Epworth Sleepiness Score	2.5 ± 3.4

Values are presented as mean ± SD.

Participants were screened using a general health questionnaire to determine their eligibility to participate in the study. Inclusion criteria were: age 20–35 years, non-smoker, non-shiftworker, caffeinated beverage consumption ≤ 120 mg/day (~ 2 cups of coffee); consumption of ≤ 2 standard alcoholic beverages/week; habitual bedtimes between 2200 and 0000 h; rise times between 0600–0800 h; absence of previous diagnosis of psychiatric and/or neurological problems; no trans-meridian travel in the previous four weeks; free from medication and drugs acting on the central nervous system, known to interfere with sleep or glucose- and/or lipid-lowering medication; and no history of habitual napping. Participants were also required to have a waist circumference < 100 cm, an Epworth Sleepiness Scale score < 10 (Johns, 1991), a global Pittsburgh Sleep Quality Index ≤ 5 (Buysse et al., 1989), a low or moderate score on the International Physical Activity Questionnaire (IPAQ Research Committee, 2005), normal scores on the 21-item Depression Anxiety Stress Scale (Lovibond and Lovibond, 1995), and moderately morning/evening or neither chronotype on the Horne-Ostberg Morningness/Eveningness Questionnaire (Horne and Ostberg, 1975).

### 2.3 Randomisation

Participants were randomised to one of two possible trial orders, 1) SIT then ACT, or 2) ACT then SIT. Three participants performed trial order 1 and three participants performed trial order 2. A computerised randomisation list of participant and order were allocated into envelopes and kept by an independent third party. The envelopes were opened once informed consent was obtained for all participants. Participants were told of their trial order upon entering the laboratory for the first trial.

### 2.4 Pre-experimental procedures

Following screening, participants attended a familiarisation visit at the research laboratory. During this visit, participants were given the opportunity to ask questions about the experimental protocol, underwent training on cognitive tests, and completed practice questionnaires. In the week prior to the study, participants were instructed to maintain their normal sleep behaviour to reduce the likelihood of sleep debt upon entering the study. To minimise the potential carry over effects of physical activity, participants were also instructed to avoid any moderate and/or vigorous physical activity for at least 48 h prior to each trial. To ensure fidelity of participants’ habitual sleep and physical activity levels and the aforementioned pre-experimental requirements, participants wore an activity monitor (Actical MiniMitter/Respironics, Bend, OR) on their non-dominant wrist and completed a sleep diary. Activity monitors are a useful and valid means for estimating total sleep time and wakefulness (Marino et al., 2013).

### 2.5 Experimental procedure

Participants lived in a sound-attenuated and temperature-controlled (21 ± 2 °C) laboratory on two separate occasions for five consecutive days and nights. On the arrival day, participants arrived at 1400 h, performed training on the cognitive performance tasks and were familiarised with walking on a motorised treadmill on a level incline. Following a baseline sleep (BL; 2200–0700 h) participants were sleep restricted for three nights (E1-E3; 0200–0700 h) with their final night a recovery night (2200–0700 h). During wake periods, light levels were maintained at 100 lx, apart from on nights E1–E3 where at 2330 h lights were dimmed to < 10 lx to avoid the phase shifting properties of light in the early biological night (Zeitzer et al., 2000).

In the SIT condition, participants remained seated for the entire study except when walking to the dining room to eat meals or to use the bathroom (32 m and 8 m from their seated position, respectively). In the ACT condition, between 1000 and 1700 h, participants rose from their seated position every 30 min and completed a 3-min bout of light-intensity walking on a motorised treadmill (Healthrider H95T; Icon Health and Fitness Inc., Utah, USA) at 3.2 km/h on a level incline (0% gradient). This process occurred 17 times, resulting in a total of 51 min of light-intensity physical activity across this 7-h period. At all other times, participants remained seated, except when walking to the dining room to eat meals or to use the bathroom. In both the SIT and ACT conditions, when not engaged in physical activity or performing cognitive or physiological testing, participants were permitted to perform quiet activities such as studying, reading or watching television. At all times, participants were monitored by researchers (either in person or via closed circuit cameras) to ensure they did not fall asleep or perform physical activity at non-specified times.

Daily energy intake was determined using a modified Harris Benedict equation (Roza and Shizgal, 1984) using an activity factor of 1.5 (Wennberg et al., 2016). Previous research has found that sleep restriction increases total daily energy expenditure at a rate of 1% per hour (Jung et al., 2011). Therefore, on E1–E3 participants’ energy intake was increased by 4% (9 h − 5 h = 4 h). Standardised meals for breakfast, lunch, and dinner were served at 0815 h, 1230 h and 1800h, respectively. Snacks were provided at 1430 h and 2330 h on BL, E1, and E2. On E3, participants received a snack only at 1430 h (as their sleep opportunity began at 2200 h that night). The macronutrient profile of daily food consumed was similar to a standard Western diet (26% protein, 20% fat, 54% carbohydrate). Participants consumed 100% of all meals. Participants were weighed each morning prior to breakfast.

### 2.6 Measures

#### 2.6.1 Steps

The number of steps performed by participants was continuously recorded using a wrist-worn receiver (M400, Polar, Kempele, Finland) and determined using a validated manufacturer propriety algorithm in the software (Polar Flow, Kempele, Finland).

#### 2.6.2 Sleep

Polysomnography, a gold standard technique for determining total sleep time (TST), was used during the experimental trial (Grael PSG/EEG Systems; Compumedics, Melbourne, Australia).

A standard montage of electrodes, including three electroencephalographic (EEG) channels (C3-M2, C4-M1, F4-O2), right and left electro-oculargraphic (EOG), and three channels of chin/lower jaw electromyographic (EMG), was applied to participants’ face and scalp in the 60 min prior to lights out. Each night of sleep was single-scored in 30 s epochs according to standard criteria by a single experienced sleep technician who was blind to the experimental conditions (Iber, 2007). TST, calculated as the total amount of sleep of any stage obtained between lights out and lights on in minutes, was obtained from each recording.

#### 2.6.3 Blood glucose

Interstitial glucose concentrations were measured using a MiniMed Continuous Glucose Monitoring System (CGMS, Medtronic Australasia PTY, Adelaide, Australia). This system has been validated against plasma glucose (Keenan et al., 2009) and has been previously used in sleep restriction studies to assess glucose concentrations (Reynolds et al., 2012). The CGMS sensor was inserted under the skin of the abdomen (5 cm to the right of the navel into the interstitial fluid) and glucose concentrations were measured every 5 min. Capillary finger pricks were administered every 5 h for calibration, using a lancing device (Accu-check Softclix Lancing Device LD01, Roche Diabetes Care Inc, United States) in conjunction with glucose electrode test strips (Optium, Roche Diabetes Care Inc, United States) and a blood glucose meter (Optium Xceed, Roche Diabetes Care Inc, United States) in accordance with the manufacturer’s instructions. The total area under the interstitial glucose response curve (total AUC) was derived using the trapezoidal method from the CGMS measurements (0700–2200 h). Daily minimum, maximum, and mean interstitial glucose concentrations were calculated from raw interstitial glucose values.

### 2.7 Cognitive and subjective test battery

During scheduled wake periods, the cognitive and subjective test battery was administered every 2 h during BL, and E1-E3 (0905 h, 1105 h, 1305 h, 1505 h, 1705h, 1905h, 2105 h, test battery bouts 1–7). Regular testing provided a comprehensive assessment of the diurnal rhythm that many of these variable exhibit. On BL and on E1–E2, two additional test batteries were performed at 2305 h and 0105 h (test battery bouts 8 and 9).

#### 2.7.1 Psychomotor vigilance test

Neurobehavioural performance was measured using a 10-min psychomotor vigilance task (PVT). This task requires participants to respond as quickly as possible to a visual stimulus appearing on a portable electronic hand-held unit (PVT-192, Ambulatory Monitoring Inc., Ardsley, NY) at random intervals of 2–10 s (Dinges and Powell, 1985). Participants were trained on this task (three practices) on the arrival day (Dinges et al., 1997). Performance on the PVT is sensitive to sleep loss and circadian misalignment (Lim and Dinges, 2008, Van Dongen et al., 2003). The number of lapses (response times > 500 ms) and mean reciprocal response times (RRT; 1/ms × 1000) were derived and used in the subsequent analyses.

#### 2.7.2 Digit symbol substitution test

The digit symbol substitution test (DSST) is a subject-paced pen and paper task that assesses attention, response speed, and visuomotor coordination. The test comprises a key consisting of numbers one to nine that are linked to nine unique predetermined symbols. Participants are given 90 s to draw the corresponding paired symbol with a set of randomly ordered numbers on the test sheet. The total number of correct responses (accurate and legible) was used as a measure of working memory performance. The key and order of numbers of the test sheet varied with each test bout. Participants were familiarised on this task on the arrival day (14 practices).

#### 2.7.3 Karolinska sleepiness scale

Subjective sleepiness was assessed using the Karolinska Sleepiness Scale (KSS) (Åkerstedt and Gillberg, 1990). Scores on the 9-point KSS Likert scale range from 1 (extremely alert) to 9 (extremely sleepy, fighting sleep). Participants were asked to rate their current level of sleepiness.

#### 2.7.4 Alertness

Subjective alertness was assessed using a 100-mm visual analogue scales (VAS). Participants were instructed to place a vertical line on the scale in response to the question “How alert do you feel right now?”. The scale was anchored with the statements ‘not at all’ and ‘completely’.

### 2.8 Statistical analyses

The statistical software package SPSS Statistics 22 (IBM Corporation) was used to analyse all data. Independent t-tests were used to examine differences in the measured variables on the baseline day (i.e., prior to the SIT or ACT intervention). A series of linear mixed-effects analysis of variance (ANOVA) models were used to determine differences in measured variables over the experimental days (E1–E3). For TST, number of steps, mean, maximum, minimum interstitial glucose, and AUC of interstitial glucose, the fixed effects were condition (SIT or ACT), day (E1–E3) and order (1, 2). To determine the impact of sleep restriction on the interstitial glucose variables irrespective of trial condition, an additional model was run which included the baseline day (BL) and experimental days (E1–E3). The fixed effects were condition (SIT or ACT), day (BL, E1–E3), and order (1 or 2). For variables in the cognitive and subjective test battery (PVT, DSST, KSS, VAS_alertness), the fixed effects were condition (SIT or ACT), day (E1–E3), test battery bout (1–9), and order (1 or 2). The random effect of participant was used to account for individual differences in all models. There were no significant main effects of order in any of the models. A Satterthwaite correction was applied to denominator degrees of freedom. Data are reported as mean ± SD, unless otherwise stated.

## 3 Results

### 3.1 Baseline day

During both trials, participants completed a baseline day prior to the SIT or ACT intervention. There were no significant differences between the SIT or ACT conditions in all measured variables at baseline (P ≥ 0.500).

### 3.2 Sleep, steps, and weight

TST and number of steps during the conditions are shown in Table 2. TST did not differ between the SIT and ACT conditions (F_1__,__36_ = 12.8, P = 0.445). However, there was a main effect of experimental day (F_1__,__36_ = 12.7, P < 0.001). Pairwise comparisons revealed that participants slept 5.5 min and 7.8 min less on E1 compared with E2 (P = 0.004) and E3 (P < 0.001), respectively. There was no difference in TST between E2 and E3 (P = 0.493).

**Table 2:** Daily measures of sleep, steps, cognitive function, subjective ratings, and interstitial glucose concentrations. Data are reported as mean ± SD.

	**Baseline day**		**Experimental day 1**		**Experimental day 2**		**Experimental day 3**	
	**Sitting**	**Active**	**Sitting**	**Active**	**Sitting**	**Active**	**Sitting**	**Active**
***Variable***								
Total sleep time (h)	7.7 ± 1.0	7.7 ± 1.4	4.7 ± 0.1	4.7 ± 0.1	4.8 ± 0.1	4.8 ± 0.1	4.9 ± 0.1	4.8 ± 0.1
Steps (count)	1129 ± 747	1128 ± 318	1303 ± 564	6991 ± 1111	1342 ± 496	6985 ± 1083	1686 ± 796	6973 ± 1377
***Interstitial glucose***								
AUC_0700-2200_ (mmol/L/900 min)	946 ± 49	995 ± 115	1081 ± 87	1128 ± 202	1063 ± 76	1106 ± 144	1035 ± 64	1104 ± 146
Daily mean (mmol/L)	5.3 ± 0.3	5.5 ± 0.9	6.0 ± 0.5	6.3 ± 1.1	5.9 ± 0.4	6.1 ± 0.4	5.7 ± 0.4	6.0 ± 0.8
Daily minimum (mmol/L)	3.7 ± 0.6	4.0 ± 0.8	5.2 ± 4.7	5.0 ± 1.0	4.7 ± 0.4	4.6 ± 0.6	4.3 ± 0.8	4.5 ± 0.6
Daily maximum (mmol/L)	7.0 ± 0.2	7.4 ± 1.2	8.0 ± 0.6	8.4 ± 1.4	8.4 ± 1.0	8.2 ± 1.3	8.5 ± 0.8	8.3 ± 1.8
***Cognitive***								
PVT - Mean reciprocal reaction time (1/RT)	4.5 ± 0.2	4.5 ± 0.7	4.4 ± 0.2	4.2 ± 0.5	4.4 ± 0.2	4.2 ± 0.5	4.4 ± 0.3	4.2 ± 0.5
PVT - Lapses (count)	0.1 ± 0.3	0.7 ± 1.3	0.3 ± 0.7	1.1 ± 1.8	0.8 ± 1.7	0.8 ± 1.0	1.4 ± 3.4	1.5 ± 3.0
DSST - Correct responses (count)	65 ± 20	65 ± 20	65 ± 19	69 ± 5	66 ± 21	72 ± 4	67 ± 22	68 ± 24
***Subjective***								
Sleepiness (1–9)	3.6 ± 2.0	3.6 ± 1.8	4.1 ±1.5	3.9 ± 1.9	4.4 ± 1.5	4.0 ± 1.7	3.8 ± 0.9	2.8 ± 1.5
Alertness (mm)	69 ± 20	67 ± 14	63 ± 15	59 ± 15	59 ± 11	59 ± 10	60 ± 7	72 ± 18

Participants performed a higher number of steps in the ACT condition compared to the SIT condition (F_1__,__36_ = 361.4, P < 0.001), however there was no main effect of day (F_2__,__36_ = 0.160, P = 0.853). Therefore, participants performed a higher number of steps in the ACT condition, compared to the SIT condition (by design) and there was no difference across the experimental days for either condition. There were no differences in the number of times participants walked to the bathroom (mean ± SD, SIT = 4.4 ± 2.5; ACT = 4.5 ± 2.4; P = 0.521) or dining room (3 times) each day between conditions.

There was no significant difference in weight prior to the intervention and each subsequent morning.

### 3.3 Interstitial glucose concentration

**Fig. 2:**
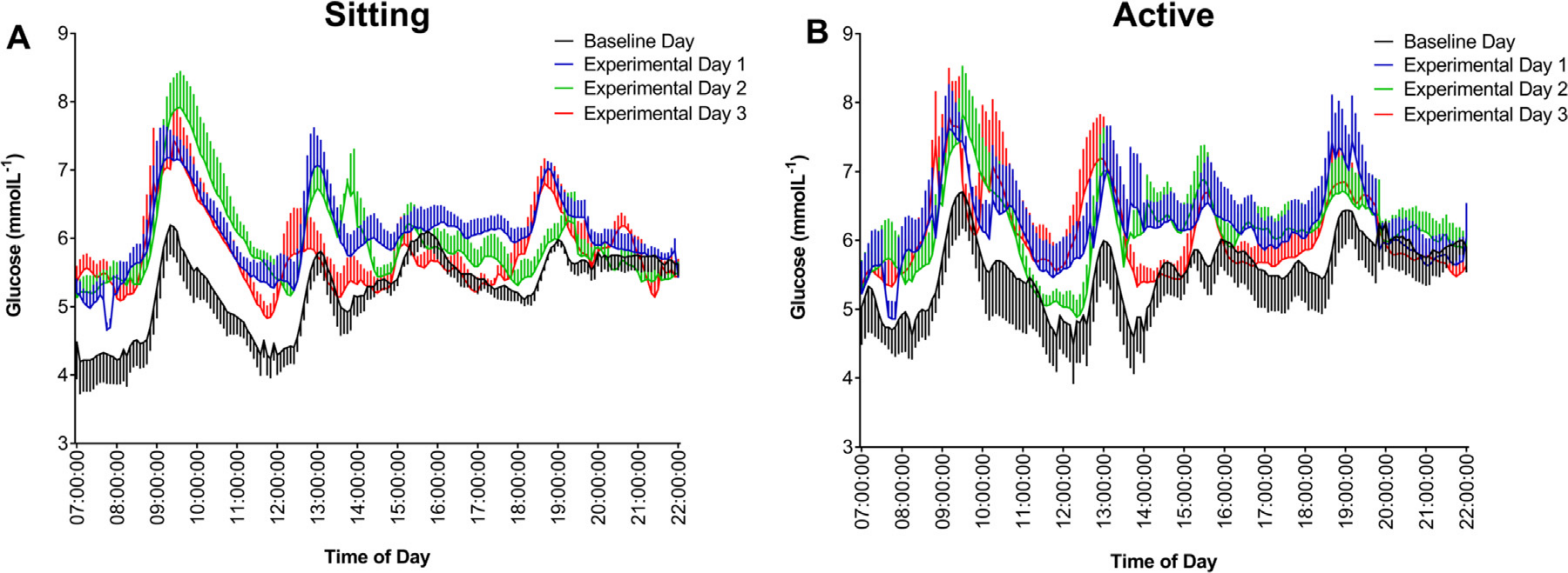
Daily interstitial glucose profiles during the sitting condition (A) and the active condition (B). Data are reported as mean ± SD.

Interstitial glucose profiles are shown in Fig. 2 and interstitial glucose variables are presented in Table 2. Minimum (F_1,36_ = 0.478, P = 0.494), mean (F_1,36_ = 1.9, P = 0.178) and maximum (F_1,36_ = 0.080, P = 0.776) interstitial glucose concentrations, and AUC (F_1,36_ = 0.274, P = 0.176) did not differ between the SIT and ACT conditions. There was no main effect of day on any of the interstitial glucose variables (P ≥ 0.298). When the baseline day was included in the analyses, there still was no main effect of condition (P ≥ 0.115), but a significant main effect of day for all variables (P ≤ 0.033). Pairwise comparisons revealed that mean interstitial glucose concentration (P = 0.038) and AUC (P = 0.038) were higher on E1 compared to BL, but were not different between BL and E2 or BL and E3 (P ≥ 0.108). Minimum interstitial glucose concentration was higher in BL compared with both E1 (P = 0.002) and E2 (P = 0.017), but there was no different between BL and E3 (P = 0.128). Maximum interstitial glucose concentration was higher in BL compared with E3 (P = 0.026), but was not different between E1 and E2 (P ≥ 0.106). In summary, interstitial glucose concentration was unaffected by the experimental conditions (SIT or ACT), but AUC, as well as mean and minimum interstitial glucose concentrations increased following the first night of sleep restriction, irrespective of condition.

### 3.4 Cognitive performance

All cognitive performance data are shown in Table 2. Mean reciprocal reaction time (F_1,300_ = 0.01, P = 0.918) and number of lapses (F_1,300_ = 0.57, P = 0.450) on the PVT, and the number of correct responses on the DSST (F_1,300_ = 0.12, P = 0.727) did not differ between the SIT and ACT conditions. There was also no main effect of day for any of these variables (P ≥ 0.443). There was a main effect of test battery bout for number of lapses (F_8,300_ = 2.7, P = 0.005), but not for mean reciprocal reaction time (F_8,300_ = 1.9, P = 0.058) or the number of correct responses (F_8,300_ = 0.54, P = 0.822). Collectively, these results indicate that there was no difference in measures of sustained vigilance or working memory performance between the SIT and ACT conditions, or between days irrespective of condition. Further, the number of lapses varied across the test battery bouts, irrespective of condition and day.

### 3.5 Subjective sleepiness and alertness

All subjective data are shown in Table 2. For subjective sleepiness, there was no main effect of day (F_2,300_ = 1.1, P = 0.321), however there was a significant main effect of condition (F_1,300_ = 8.06, P = 0.005) and test battery bout (F_8,300_ = 8.7, P < 0.001). These results indicate higher levels of subjective sleepiness for the SIT condition compared to the ACT condition, and a build-up of subjective sleepiness across the day regardless of condition or day. There was no condition by test battery bout interaction (F_8,300_ = 0.22, P = 0.986).

For subjective alertness, there was a main effect of condition (F_1,300_ = 10.6, P < 0.001), day (F_2,300_ = 9.3, P < 0.001), test battery bout (F_8,300_ = 8.7, P < 0.001), but no interactions were observed between condition by day (F_2,300_ = 2.5, P = 0.081) or condition by test bout (F_8,300_ = 0.18, P = 0.994). Specifically, subjective alertness was higher in the ACT condition compared with the SIT condition, and subjective alertness decreased across test bouts and experimental days irrespective of condition.

## 4 Discussion

This study is the first to report on the impact of breaking up prolonged sitting with light-intensity walking breaks on measures of glucose metabolism and cognitive function under conditions of sleep restriction. The major finding of this pilot study was that when sedentary, healthy, male adults were sleep restricted, breaking up prolonged sitting with light-intensity walking breaks did not benefit glucose metabolism. Another important finding was that while breaking up prolonged sitting with light-intensity walking breaks did not improve measures of cognitive performance, participants reported feeling less sleepy and more alert, compared to when sitting was uninterrupted.

Prospective experimental studies provide considerable evidence of the positive effects of breaking up prolonged sitting on metabolic health outcomes (Chastin et al., 2015). However, a potential limitation of these studies is that the impact of prior sleep duration (or sleep restriction) was not considered. In accordance with previous research (Buxton et al., 2010, Spiegel et al., 1999), we found that, irrespective of experimental condition, glucose metabolism measures were elevated following the first night of sleep restriction when compared to the baseline day (9-h sleep opportunity). However, no improvement was observed in measures of interstitial glucose when prolonged sitting was broken up during subsequent days of sleep restriction. Our results suggest that the increased muscular contraction caused by breaking up prolonged sitting with physical activity—which is thought to mediate glucose uptake and increase insulin sensitivity (Bergouignan et al., 2016) in non-sleep restricted individuals—may not be replicated under conditions of sleep restriction. Future studies are needed to determine whether sleep restriction offsets or attenuates the benefits of breaking up sitting. This could be achieved by assessing how different combinations of activity (e.g., sitting versus active) and sleep (e.g., 5-h time in bed versus 9-h time in bed) acutely affect markers of cardiometabolic health. Such studies would also determine whether increasing an individual’s sleep in combination with breaking up periods of prolonged sitting could further reduce cardiometabolic risk, i.e., the combined effect of adequate sleep and breaking up prolonged sitting is stronger than either alone. If future studies show that sleep restriction offsets the benefits of breaking up prolonged sitting, then sleep-restricted individuals may not benefit as much, or perhaps at all, from breaking up prolonged sitting. Therefore, public health recommendations relating to breaking up prolonged sitting may also need to include a statement with regards to hours of sleep required.

Measures of cognitive performance, specifically sustained attention and working memory, were not affected by breaking up prolonged sitting. However, the light-intensity walking breaks improved self-reported levels of sleepiness and alertness. While the literature examining breaking up prolonged sitting on cognitive and subjective performance in healthy adults is sparse (Wennberg et al., 2016), our results are supported by previous research that has assessed the impact of physical activity on these measures (Horne and Foster, 1995, LeDuc et al., 2000, Sallinen et al., 2008). Some studies have found that following sleep restriction, low- and high-intensity physical activity improved levels of alertness in the short-term (10–30 min) (Horne and Foster, 1995, LeDuc et al., 2000), however there were no corresponding improvements in measures of cognitive, work performance (LeDuc et al., 2000), or vigilance (Horne and Foster, 1995). To counteract the induced impairments in alertness and cognitive functioning following sleep restriction, Sallinen et al. (2008) investigated the efficacy of regular light neck and shoulder exercises during rest breaks, as a practical alternative in work environments (e.g. compared with treadmill or bicycle exercise). The light-intensity physical activity was not an effective countermeasure for cognitive functioning following sleep restriction, however, self-reported sleepiness was improved when compared to a control condition with no rest break (Sallinen et al., 2008). Attenuations in fatigue levels have been observed in office workers who transitioned from a seated to a standing work posture every 30 min, relative to seated-only work (Thorp et al., 2014). Further, using the same protocol as the current study, Wennberg and colleagues found that intermittent light-intensity walking breaks resulted in an attenuation of subjective fatigue levels but no changes in cognitive performance (Wennberg et al., 2016). Taken in conjunction with previous findings, our results suggest that while individuals' sleepiness and alertness were improved, breaking up prolonged sitting with light-intensity walking breaks does not appear to act as a countermeasure for the acute cognitive deficits associated with sleep restriction.

A strength of this study was the use of a crossover counterbalanced experimental design with a well-controlled protocol. However, given this was a pilot study it may be underpowered and the characteristics of participants (i.e., young sedentary healthy males) may not be generalisable to other populations (e.g., women, older adults, clinical populations). Further, given our sample size, we were not able to investigate whether the observed effects are moderated by participant characteristics (e.g., age, level of fitness, diet). While our results provide the first evidence that breaking up sitting with light-intensity walking may not benefit markers of cardiometabolic health under conditions of sleep restriction, it is possible that the intensity of the physical activity was too low, the duration too short, and/or the frequency of sessions too few to elicit changes in glucose metabolism. In addition, while acute studies are important to establish the potential mechanisms of action through which sleep restriction and breaking up prolonged sitting interact, the long-term impacts cannot be elucidated from this study. A recent systematic review concluded that more research was required to determine the type, intensity, duration, and frequency of physical activity necessary to counteract the detrimental effects of prolonged sitting, and also highlighted that these effects may differ according to participant characteristics (e.g., weight status, fitness level) (Benatti and Ried-Larsen, 2015). As previously mentioned future studies should include more rigorous assessments of glucose metabolism (e.g., oral glucose tolerance tests, measures of insulin sensitivity). Further, future multicomponent interventions may be needed that involve promoting a mix of behaviours (e.g., regular exercise, breaking up sitting, a healthy diet, adequate sleep) to benefit health (Chastin et al., 2015).

In summary, breaking up prolonged sitting with light-intensity walking breaks under conditions of sleep restriction did not impact upon acute measures of glucose metabolism. Further, intermittent light-intensity walking breaks resulted in the attenuation of self-reported sleepiness and improved alertness when compared to uninterrupted sitting. However, these differences did not translate to improved cognitive performance.

## References

[bib1] Adams R., Appleton S., Taylor A., McEvoy D., Antic N. (2016). Report to the Sleep Health Foundation, 2016 Survey of Australian Adults.

[bib2] Åkerstedt T., Gillberg M. (1990). Subjective and objective sleepiness in the active individual. Int. J. Neurosci..

[bib3] Bankoski A. (2011). Sedentary activity associated with metabolic syndrome independent of physical activity. Diabetes Care.

[bib4] Bauman A.E., Chau J.Y., Ding D., Bennie J. (2013). Too much sitting and cardio-metabolic risk: an update of epidemiological evidence. Curr. Cardiovasc. Risk Rep..

[bib5] Belenky G. (2003). Patterns of performance degradation and restoration during sleep restriction and subsequent recovery: a sleep dose-response study. J. Sleep Res..

[bib6] Benatti F.B. (2017). Intermittent standing but not a moderate exercise Bout reduces postprandial glycaemia. Med. Sci. Sport. Exerc..

[bib7] Benatti F.B., Ried-Larsen M. (2015). The effects of breaking up prolonged sitting time: a review of experimental studies. Med. Sci. Sport. Exerc..

[bib8] Bergouignan A. (2016). Frequent interruptions of sedentary time modulates contraction-and insulin-stimulated glucose uptake pathways in muscle: ancillary analysis from randomized clinical trials. Sci. Rep..

[bib9] Broussard J.L., Ehrmann D.A., Van Cauter E., Tasali E., Brady M.J. (2012). Impaired insulin signaling in human adipocytes after experimental sleep restriction: a randomized, crossover study. Ann. Int. Med..

[bib10] Buxton O.M., Pavlova M., Reid E.W., Wang W., Simonson D.C., Adler G.K. (2010). Sleep restriction for 1 week reduces insulin sensitivity in healthy men. Diabetes.

[bib11] Buysse D.J., Reynolds C.F., Monk T.H., Berman S.R., Kupfer D.J. (1989). The Pittsburgh sleep quality index: a new instrument for psychiatric practice and research. Psychiatry Res..

[bib12] Cannon C.P. (2007). Cardiovascular disease and modifiable cardiometabolic risk factors. Clin. Cornerstone.

[bib13] Centers for Disease Control Prevention (2011). Unhealthy Sleep-related Behaviors 12 States, 2009. MMWR Morbidity and Mortality Weekly Report.

[bib14] Chang Y.-K. (2015). Dose-response relation between exercise duration and cognition. Med. Sci. Sport. Exerc..

[bib15] Chastin S.F., Palarea-Albaladejo J., Dontje M.L., Skelton D.A. (2015). Combined effects of time spent in physical activity, sedentary behaviors and sleep on obesity and cardio-metabolic health markers: a novel compositional data analysis approach. PLoS One.

[bib16] Dinges D., Powell J.W. (1985). Microcomputer analyses of performance on a portable, simple visual RT task during sustained operations. Behav. Res. Methods Instrum. Comput..

[bib17] Dinges D.F. (1997). Cumulative sleepiness, mood disturbance, and psychomotor vigilance performance decrements during a week of sleep restricted to 4–5 h per night. Sleep.

[bib18] Hoevenaar-Blom M.P., Spijkerman A., Kromhout D., van den Berg J.F., Verschuren W. (2011). Sleep duration and sleep quality in relation to 12-year cardiovascular disease incidence. Sleep.

[bib19] Horne J., Foster S. (1995). Can exercise overcome sleepiness. Sleep Res..

[bib20] Horne J.A., Ostberg O. (1975). A self-assessment questionnaire to determine morningness-eveningness in human circadian rhythms. Int. J. Chronobiol..

[bib21] Iber C. (2007). The AASM manual for the scoring of sleep and associated events: rules, terminology and technical specifications. Am. Acad. Sleep Med..

[bib22] IPAQ Research Committee, 2015. Guidelines for data processing and analysis of the International Physical Activity Questionnaire (IPAQ)–short and long forms, vol. 17, p. 2008.

[bib23] Johns M.W. (1991). A new method for measuring daytime sleepiness: the Epworth sleepiness scale. Sleep.

[bib24] Jung C.M., Melanson E.L., Frydendall E.J., Perreault L., Eckel R.H., Wright K.P. (2011). Energy expenditure during sleep, sleep deprivation and sleep following sleep deprivation in adult humans. J. Physiol..

[bib25] Katzmarzyk P.T. (2014). Standing and mortality in a prospective cohort of Canadian adults. Med. Sci. Sport. Exerc..

[bib26] Keenan D.B., Mastrototaro J.J., Voskanyan G., Steil G.M. (2009). Delays in minimally invasive continuous glucose monitoring devices: a review of current technology. J. Diabetes Sci. Technol..

[bib27] Knutson K.L. (2010). Sleep duration and cardiometabolic risk: a review of the epidemiologic evidence. Best Pract. Res. Clin. Endocrinol. Metab..

[bib28] Koster A. (2012). Association of sedentary time with mortality independent of moderate to vigorous physical activity. PLoS One.

[bib29] LeDuc P.A., Caldwell J.A., Ruyak P.S. (2000). The effects of exercise as a countermeasure for fatigue in sleep-deprived aviators. Mil. Psychol..

[bib30] Lim J., Dinges D.F. (2008). Sleep deprivation and vigilant attention. Ann. N.Y. Acad. Sci..

[bib31] Lovibond P.F., Lovibond S.H. (1995). The structure of negative emotional states: comparison of the Depression Anxiety Stress Scales (DASS) with the Beck Depression and Anxiety Inventories. Behav. Res. Ther..

[bib32] Marino M. (2013). Measuring sleep: accuracy, sensitivity, and specificity of wrist actigraphy compared to polysomnography. Sleep.

[bib33] Nedeltcheva A.V., Kessler L., Imperial J., Penev P.D. (2009). Exposure to recurrent sleep restriction in the setting of high caloric intake and physical inactivity results in increased insulin resistance and reduced glucose tolerance. J. Clin. Endocrinol. Metab..

[bib34] Peddie M.C., Bone J.L., Rehrer N.J., Skeaff C.M., Gray A.R., Perry T.L. (2013). Breaking prolonged sitting reduces postprandial glycemia in healthy, normal-weight adults: a randomized crossover trial. Am. J. Clin. Nutr..

[bib35] Reynolds A.C. (2012). Impact of five nights of sleep restriction on glucose metabolism, leptin and testosterone in young adult men. PLoS One.

[bib36] Roza A.M., Shizgal H.M. (1984). The Harris Benedict equation reevaluated: resting energy requirements and the body cell mass. Am. J. Clin. Nutr..

[bib37] Sallinen M. (2008). Recovery of cognitive performance from sleep debt: do a short rest pause and a single recovery night help?. Chronobiol. Int..

[bib38] Schmid S.M., Hallschmid M., Schultes B. (2015). The metabolic burden of sleep loss. Lancet Diabetes Endocrinol..

[bib39] Spiegel K., Leproult R., Van Cauter E. (1999). Impact of sleep debt on metabolic and endocrine function. Lancet.

[bib40] Thorp A.A., Kingwell B.A., Owen N., Dunstan D.W. (2014). Breaking up workplace sitting time with intermittent standing bouts improves fatigue and musculoskeletal discomfort in overweight/obese office workers. Occup. Environ. Med..

[bib41] Thorp A.A., Owen N., Neuhaus M., Dunstan D.W. (2011). Sedentary behaviors and subsequent health outcomes in adults: a systematic review of longitudinal studies, 1996–2011. Am. J. Prev. Med..

[bib42] Tremblay M.S. (2016). Canadian 24-h movement guidelines for children and youth: an integration of physical activity, sedentary behaviour, and sleep 1. Appl. Physiol. Nutr. Metab..

[bib43] Van Dongen H.P.A., Maislin G., Mullington J.M., Dinges D.F. (2003). The cumulative cost of additional wakefulness: dose-response effects on neurobehavioral functions and sleep physiology from chronic sleep restriction and total sleep deprivation. Sleep.

[bib44] Watson N. (2015). Recommended amount of sleep for a healthy adult: a joint consensus statement of the American academy of sleep medicine and sleep research society. J. Clin. Sleep Med.: JCSM: Off. Pub. Am. Acad. Sleep Med..

[bib45] Wennberg P. (2016). Acute effects of breaking up prolonged sitting on fatigue and cognition: a pilot study. BMJ Open.

[bib46] Wijndaele K. (2014). Increasing objectively measured sedentary time increases clustered cardiometabolic risk: a 6 year analysis of the ProActive study. Diabetologia.

[bib47] Wilmot E.G. (2012). Sedentary time in adults and the association with diabetes, cardiovascular disease and death: systematic review and meta-analysis. Diabetologia.

[bib48] Zeitzer J.M., Dijk D.J., Kronauer R.E., Brown E.N., Czeisler C.A. (2000). Sensitivity of the human circadian pacemaker to nocturnal light: melatonin phase resetting and suppression. J. Physiol..

